# New Treatment Strategies in Advanced Epidermal Growth Factor Receptor-Driven Non-Small Cell Lung Cancer: Beyond Single Agent Osimertinib

**DOI:** 10.3390/cancers17050847

**Published:** 2025-02-28

**Authors:** Paolo Maione, Valentina Palma, Giuseppina Pucillo, Cesare Gridelli

**Affiliations:** 1 Division of Medical Oncology, S.G. Moscati Hospital, 83100 Avellino, Italy; pmaione@libero.it; 2 Division of Medical Oncology, S.G. Moscati Hospital, Università degli Studi della Campania “Luigi Vanvitelli”, 83100 Avellino, Italy; valepal.87.v@gmail.com (V.P.); giuseppinapucillo92@gmail.com (G.P.)

**Keywords:** EGFR mutant NSCLC, osimertinib, amivantamab, lazertinib, chemotherapy

## Abstract

Osimertinib has been the standard treatment for advanced Epidermal Growth Factor Receptor (EGFR)-driven non-small cell lung cancer (NSCLC) for many years. However, even with remarkable response rate, progression-free survival (PFS) and survival benefit as compared to the old generation EGFR tyrosine kinase inhibitors (TKIs) gefitinib and erlotinib, treatment outcomes for these subsets of patients remain a challenge. Recently, in order to go beyond osimertinib, new treatment strategies have been developed. In particular, in the FLAURA 2 phase III randomized trial, the combination of platin-based chemotherapy and osimertinib showed impressive PFS benefits as compared to single-agent osimertinib. Furthermore, in the MARIPOSA phase III randomized study, the combination of the anti-EGFR and anti-MET monoclonal antibody amivantamab combined with the new anti-EGFR TKI lazertinib demonstrated remarkable PFS benefit as compared to single agent osimertinib. This paper will discuss these new treatment options and potential selection criteria for personalized treatment of patients.

## Simple Summary

Patients with metastatic lung cancer harboring epidermal growth factor receptor mutations represent a very relevant subgroup of lung cancer patients because they are never smokers in the majority of the cases and for very good therapeutic outcomes. The standard treatment for these patients is represented by an oral drug, osimertinib, that offers patients both relevant survival prolongment and a very good safety profile. Very recently, new treatment strategies, including intravenous chemotherapy combined with osimertinib or the new drug amivantamab (an intravenous or subcutaneous monoclonal antibody) combined with lazertinib (an oral drug with the same mechanism of action as osimertinib) have produced in randomized trials better efficacy outcomes than osimertinib alone. In this manuscript, we try to discuss the new treatment options available for this patient population, describing their relevant efficacy outcomes and considering safety profile issues compared to the oral standard treatment, yet represented by osimertinib.

## 1. Introduction

In about 15 to 50% of patients with advanced non-squamous non-small cell lung cancer (NSCLC), tumors harbor activating mutations in the epidermal growth factor receptor gene (EGFR), with relevant clinical and therapeutic implications that make this patient population peculiar [1,2]. The most clinically influent and also more frequent EGFR mutations are exon 19 deletions (Ex19del) or exon 21 codon p.Leu858Arg (L858R) substitutions, which together account for 85 to 90% of all EGFR mutations [3]. First,- and second-generation EGFR-tyrosine kinase inhibitors (TKIs), i.e., gefitinib, erlotinib, and afatinib, have been a very effective standard first-line treatment for patients with advanced EGFR mutant NSCLC for many years, producing a relevant improvement in progression-free survival (PFS) compared with chemotherapy [4,5,6]. Also, a meta-analysis of phase III randomized trials on the comparison between first-generation EGFR TKIs (gefitinib and erlotinib) and chemotherapy as first-line treatment has confirmed a wide advantage in favor of TKIs in terms of PFS [7]. For about 5 years, the standard first-line treatment of advanced NSCLC harboring EGFR mutations, specifically Ex19del and L858R, has further changed and improved with the approval of osimertinib, an oral third-generation, irreversible but selective, EGFR–TKI [8,9]. In fact, the phase III randomized trial named FLAURA has demonstrated that osimertinib achieves better PFS and overall survival (OS) outcomes compared with the first-generation EGFR TKIs gefitinib and erlotinib [10,11]. Thereafter, other third-generation EGFR-TKIs demonstrated activity in this setting, and lazertinib has been approved as a single agent in South Korea [12,13]. Although clinical outcomes offered by osimertinib treatment have to be considered excellent in terms of efficacy and safety profile, nearly all patients experience disease progression and ultimately die of lung cancer because of resistance to treatment. Multiple mechanisms of resistance to osimertinib have been recognized, with the most common being alterations in the MET gene [14] and EGFR pathways [15,16], but about half of the patients whose tumors develop unidentified mechanisms of resistance to osimertinib [17]. Thus, the search for improvements in efficacy outcomes is mandatory in this patient population, especially for subgroups of patients with poor prognosis disease. Two phase III randomized trials, FLAURA 2 and Mariposa [18,19], have recently produced clinically relevant data on this topic that open new perspectives and possibilities in the treatment algorithms for EGFR mutant NSCLC patients (Table 1 and Table 2).

## 2. Osimertinib, the Current Valuable Standard First-Line Treatment

The phase III randomized trial named Flaura, established osimertinib, a third-generation EGFR-TKI, a few years ago as the new standard treatment of advanced EGFR mutI confirmant NSCLC over first-generation EGFR-TKIs [10,11]. In this trial, 556 patients were randomly assigned and treated with osimertinib (279 patients) or with erlotinib or gefitinib (277 patients). Osimertinib achieved a better outcome in terms of median progression-free survival compared with the previous standard treatment (18.9 months versus 10.2 months). Osimertinib performed better also in terms of response rate compared with standard EGFR-TKIs (80% versus 76%, respectively) and disease-control rate (97% versus 92%, respectively). The Flaura trial also demonstrated a better outcome in terms of overall survival in favor of osimertinib compared with old-generation TKIs. In fact, the median overall survival for patients treated with osimertinib resulted in 38.6 months, while it resulted in 31.8 months for those treated with erlotinib or gefitinib (hazard ratio for death, 0.80; *p* = 0.046). Also, survival rates at different time points (12, 24, and 36 months) resulted in improvement for patients treated with osimertinib compared with old-generation EGFR-TKIs. Moreover, the improvement in survival outcomes achieved with osimertinib treatment was extended to all the subgroups of patients enrolled in the trial. Another equally relevant point of the additional benefit associated with osimertinib treatment was the safety profile, clearly better for osimertinib compared with old-generation EGFR-TKIs, especially in terms of skin toxicity (rash or acne, 58% versus 78%). Adverse events of grade 3 or higher were reported in 34% of patients treated with osimertinib and in 45% of those treated with old-generation EGFR-TKIs. In everyday clinical practice, advanced EGFR mutant NSCLC patients are often treated with radiotherapy simultaneously or sequentially to osimertinib for localized progressive disease (e.g., metastases in bone or lymph nodes, progressive primary tumor, etc.). This approach in oligo-progressive disease is often successful and leads to prolonged treatment with osimertinib and to delay of second-line systemic treatment. However, attention must be paid to safety issues, especially if radiation is applied to the thorax while patients are on osimertinib, with a high rate of severe radiation pneumonitis reported in two retrospective studies [20,21]. In conclusion, with the availability of the third-generation EGFR-TKI osimertinib, patients with advanced EGFR mutant NSCLC have been and are being treated with further improvement in efficacy outcomes and with a globally better safety profile leading intuitively to a better quality of life for patients.

## 3. Beyond Single-Agent Osimertinib as First-Line Treatment

### 3.1. Platin-Based Chemotherapy Plus Osimertinib

#### 3.1.1. Efficacy

The attempt to improve the outcomes of EGFR-TKIs by combining them with platin-based chemotherapy has already been successfully tested in phase II and III trials for the first-generation EGFR-TKI gefitinib. In fact, gefitinib combined with chemotherapy with carboplatin and pemetrexed performed better than gefitinib alone [22,23,24,25]. Thus, this type of approach has also been tested for osimertinib, the current standard treatment for patients with advanced EGFR mutant NSCLC, and recently culminated in a very relevant phase III randomized trial named FLAURA 2. Precisely, the FLAURA 2 trial was developed before in a safety run-in phase aimed at confirming that the safety profile of the combination was mild enough and predictable to challenge the subsequent phase III of the trial. This safety run-in phase was successfully completed with positive results that allowed phase III of the trial to be developed [26]. Thus, patients were subsequently randomized in a 1:1 ratio to be treated with osimertinib plus platin-based chemotherapy or with osimertinib alone. In both treatment arms, osimertinib was administered at the dose of 80 mg once daily. In the combination arm, patients were also treated with cisplatin or carboplatin (at the investigator’s discretion) plus pemetrexed. Moreover, in the combination arm, after four cycles of platin-based chemotherapy, patients had the possibility to continue maintenance treatment with chemotherapy (pemetrexed) plus osimertinib. The number of patients randomized (557) was interestingly almost the same as the FLAURA 1 trial, with 279 patients treated with chemotherapy plus osimertinib and 278 patients treated with osimertinib alone. The experimental arm, chemotherapy plus osimertinib, produced statistically significant better outcomes in terms of overall investigator-assessed PFS, the primary endpoint of the study. In fact, the median PFS for patients treated with the combination was 25.5 months versus 16.7 months for patients treated with osimertinib alone (HR = 0.62; *p* < 0.001). It is very interesting to underline that PFS curves separated early in favor of the combination arm and not after months. The improvement in PFS achieved with the combination of chemotherapy plus osimertinib was reported in all the subgroups of patients, including differences in mutation type and presence or absence of brain metastases. It is very interesting for future clinical speculation and decision-making the evidence that the difference in performance favoring the combination was wider for patients whose tumors harbored L858R mutation (24.7 versus 13.9 months) compared with those harboring exon 19 deletion (27.9 months versus 19.4 months). Equally of interest is the evidence that the benefit achievable with the combination chemotherapy plus osimertinib seems to be of higher degree for patients with brain metastases at the beginning of the trial (median PFS 24.9 with the combination versus 13.8 months with osimertinib alone) compared with patients without brain metastases (PFS 27.6 months versus 21.0 months, respectively). Moreover, in patients with the presence of brain metastases at baseline, the combination of chemotherapy and osimertinib also achieved a longer central nervous system (CNS) median PFS time and a higher rate of complete responses in CNS lesions, compared with osimertinib alone [27]. In terms of global antitumor activity, the combination produced a slightly higher response rate compared with osimertinib alone (83% versus 76%) but reported a longer median duration of response (24.0 versus 15.3 months, respectively). Overall survival was a secondary endpoint, and data are not mature yet; however, a second interim analysis has been recently presented, showing a positive trend towards a median OS benefit in favor of the combination compared with osimertinib alone in the Asian cohort of the trial (40.5 versus 38.7 months) [28]. Very recently, another subgroup analysis of the FLAURA 2 trial has been reported, showing a further enhanced PFS benefit in favor of the combination of chemotherapy plus osimertinib versus osimertinib alone in patients with a poor prognostic factor as the high tumor burden, defined in this analysis as the presence at baseline of three or more metastatic sites, compared with the benefit observed in those patients whose tumors had less than three metastatic sites [29].

#### 3.1.2. Safety

Although more effective in terms of PFS and producing a higher antitumor activity, in the FLAURA 2 trial, the combination of platin-based chemotherapy and osimertinib resulted in more toxic, as expected, than osimertinib alone. In fact, the rate of adverse events of grade 3 or higher in the group of patients treated with the combination was 64%, versus 27% in the group of patients treated with osimertinib alone. This discrepancy was mainly due, as predictable, to the unbalance of hematologic toxicity, which occurred in 71% of patients randomized to receive the combination and only 24% of those randomized to osimertinib alone. Although more toxic, patients randomized to the combination treatment experienced the discontinuation of osimertinib due to adverse events only in 11% of cases versus 6% of those treated with osimertinib alone. As a further positive comment to these interesting data, the authors of the FLAURA 2 study underline that the median PFS of patients enrolled in FLAURA and FLAURA 2 trials and treated with osimertinib alone was very similar, suggesting that also the two patient populations were similar. More precisely, the patients enrolled in the FLAURA 2 trial presented a rate of brain metastases higher than those enrolled in FLAURA (41% versus 21%), and this evidence may be relevant considering the higher benefit shown by the combination in patients with brain metastases at baseline.

### 3.2. Amivantamab Plus Lazertinib as First-Line Treatment

#### 3.2.1. Efficacy

Amivantamab is an EGFR-MET bispecific antibody with multiple mechanisms of action. In fact, it exerts its action by ligand blocking, receptor degradation, and immune actions by recruitment of effector cells as natural killer cells [30,31,32]. Its clinical development in EGFR mutant NSCLC is not by chance; in fact, amivantamab binds to EGFR extracellularly, bypassing intracellular mutations (including those at the TKI catalytic domain), and moreover, its action against MET activity aims at reacting against mechanisms of resistance to EGFR-TKIs. The first approval for the use of amivantamab has been in the treatment of patients with EGFR exon 20 insertion mutations whose disease progressed on or after platinum-based chemotherapy [33]. Moreover, amivantamab has shown activity against a wide range of activating and resistance mutations in EGFR-mutated NSCLC and in patients with MET exon 14 skip mutations [34,35]. Lazertinib, as osimertinib, is a selective, oral third-generation EGFR-TKI, effective in EGFR mutant NSCLC both with activating and T790M resistance mutations [36,37]. Thus, it is intuitive that combining amivantamab and lazertinib means targeting both the extracellular and intracellular catalytic EGFR domains, and this mechanism has already been demonstrated to produce a synergistic clinical benefit [38,39,40]. Consequently, the authors of the phase III randomized trial named MARIPOSA [19] mainly based their scientific rationale on testing the combination of amivantamab plus lazertinib as first-line treatment of advanced EGFR mutant NSCLC on the attempt to proactively target mechanisms of resistance to osimertinib [14,41]. Also in the MARIPOSA trial were enrolled patients whose tumors harbored exon 19 deletion or L858R mutations, and patients were randomized in a 2:2:1 ratio to be treated with intravenous amivantamab plus lazertinib, osimertinib alone, or lazertinib alone. The patient population was larger than FLAURA trials, with 1074 patients randomized (429 to amivantamab–lazertinib, 429 to osimertinib, and 216 to lazertinib). Intravenous amivantamab was administered as usual for this drug, i.e., weekly for the first four weeks and then every two weeks. The combination of amivantamab plus lazertinib achieved a significantly longer median PFS compared with osimertinib alone (primary endpoint of MARIPOSA trial; 23.7 versus 16.6 months, respectively, with an HR of 0.70 and *p* < 0.001). The median progression-free survival in the lazertinib group was 18.5 months (this arm was requested in the trial only to understand the contribution of the components of the experimental combination). It is to underline that in the MARIPOSA trial, differently from the FLAURA 2 trial, PFS curves did not separate early but only at 6 months. This evidence is to be remembered when comparing these new first-line options for EGFR mutant NSCLC alternatives to osimertinib alone and possibly when we will have the chance to choose among them. On the contrary, also in the MARIPOSA trial as in the FLAURA 2 trial, PFS benefit in favor of the combination (amivantamab plus lazertinib) was higher in subgroups of patients with some of the recognized poor prognosis clinical and or molecular features, i.e., patients whose tumors harbored TP53 co-mutations, patients without circulating tumor DNA clearance at cycle 3 day 1, and patients with liver metastases [42]. It is stimulating for future discussion that the combination of amivantamab and lazertinib would seem to be of higher benefit for some high-risk EGFR mutant tumors different from other poor-prognosis EGFR mutant NSCLC that would seem to be more adequately treated with the FLAURA 2 combination of platin-based chemotherapy and osimertinib (i.e., patients with brain metastases and patients whose tumors harbor L858R mutation). Another very interesting evidence and point of discussion is that the combination of amivantamab plus lazertinib was not more active in terms of response rate compared with osimertinib alone (86% versus 85%, respectively), but with a higher median duration of response (25.8 versus 16.8 months, respectively). This type of pattern of difference in antitumoral response is similar to that observed in the FLAURA 2 trial for the combination of chemotherapy plus osimertinib, but perhaps for different reasons and mechanisms (proactive treatment of resistance in MARIPOSA, killing of different cellular clones in FLAURA 2). Survival data of the MARIPOSA trial are not mature enough to draw conclusions on a possible survival benefit gained with the combination of amivantamab and lazertinib. In fact, in the interim analysis for overall survival, a not statistically significant benefit emerged in favor of the combination, with the rates of alive patients at 24 months resulted in 74% and 69% in the amivantamab plus lazertinib group and osimertinib group, respectively (HR = 0.80; 95% CI, 0.61 to 1.05). Very recently (on 7 January 2025), a press release has been announced that the combination of amivantamab plus lazertinib within the Mariposa trial has met the final pre-specified secondary endpoint of OS, demonstrating clinically meaningful and statistically significant improvement in OS (expected to exceed one year) versus the current standard of care osimertinib, but these data have not yet been published nor presented at congresses.

#### 3.2.2. Safety

As in the FLAURA 2 trial, the combination (in MARIPOSA of amivantamab and lazertinib) resulted in more effectiveness in terms of PFS as more toxic and predictable. The combination resulted in a worse safety profile in terms of the rate of grade 3 or higher adverse events, mainly paronychia, and skin rash (75% versus 43% for the combination of amivantamab plus lazertinib and osimertinib alone, respectively). The topic of infusion-related reactions and venous thromboembolic adverse events typically associated with amivantamab administration was confirmed in the MARIPOSA trial, occurring in 63% and 37% of the patients treated with amivantamab–lazertinib, respectively. However, it is to underline that the majority of infusion reactions occurred during the first infusion and the majority of thromboembolic events during the first 4 months, suggesting that a proactive approach in the clinical practice may reduce these adverse events in the future (i.e., increased use of prophylactic steroids at first infusions and prophylactic anticoagulation). Specifically, a very recent phase II trial named SKIPirr showed that prophylactic treatment with dexamethasone 8 mg oral BID during the two days preceding cycle 1 day 1 and 1 h prior to infusion of amivantamab on cycle 1 day 1 resulted in an about 3-fold reduction in infusion-related reactions rate compared with standard management (from 67.4% to 22.5%) [43]. Another relevant safety data to consider in the future if we will have the possibility to use amivantamab plus lazertinib as a first-line option is the rate of discontinuation of all trial agents resulted in 10% for patients treated with the combination and 3% for those treated with osimertinib alone (most common adverse events leading to discontinuation being infusion-related reactions and paronychia). This rate of discontinuation is very similar to that observed in the FLAURA 2 trial, where the rate of discontinuation of all trial agents resulted in 11% for the combination of platin-based chemotherapy plus osimertinib and 6% for osimertinib alone. Also, this indirect comparative safety data among these two new combinations is to be considered in the future when choosing among the three first-line options possibly available (osimertinib alone, platin-based chemotherapy plus osimertinib, amivantamab plus lazertinib), but the amivantamab plus lazertinib toxicity must also be evaluated considering the new subcutaneous formulation that reduces the toxicity profile (see later). Moreover, very recently, also some patient-reported outcomes have been reported from the MARIPOSA trial, showing that the amivantamab plus lazertinib combination significantly delayed symptomatic progression compared to osimertinib without negatively impacting patients’ functioning or health-related quality of life [44].

## 4. Amivantamab Plus Chemotherapy as Second-Line Treatment

### 4.1. Efficacy

The advances in the treatment of advanced EGFR mutant NSCLC, as discussed above, are mainly extended to first-line treatment but not exclusively. Also, in the second-line treatment, some very relevant data have been published with clinical implications that intersect with data from first-line treatment for the future building of treatment algorithms. Until osimertinib was considered the only first-line treatment option (before FLAURA 2 and MARIPOSA trial), and also nowadays in all cases, osimertinib is used as first-line treatment, the standard second-line treatment of this patient population is platinum-based chemotherapy [9,45] with PFS outcomes of 4.4–5.5 [46]. Thus, improving these dismal outcomes is also considered an unmet need for this patient population. Also, in this clinical context, amivantamab represents the option in the most advanced stage of clinical development in two types of administration, combined with chemotherapy or both chemotherapy and EGFR-TKIs. The action of amivantamab against EGFR and MET-based resistance mechanisms to osimertinib treatment [39] and the activity of platin-based chemotherapy against other resistance mechanisms that are EGFR- and MET-independent make the combination of chemotherapy and amivantamab the best candidate to be tested on or after progression to osimertinib therapy. Two preliminary trials have separately tested the two combinations (amivantamab plus chemotherapy and amivantamab plus chemotherapy plus lazertinib), both in EGFR mutant NSCLC after treatment and progression with EGFR-TKIs and showing promising antitumor activity with a safety profile allowing further phase III evaluation [47,48]. In the CHRYSALIS-2 trial (cohort A), the combination of amivantamab plus lazertinib has yielded a promising antitumor activity (overall response rate of 28%) in 162 patients with EGFR exon 19 deletion- or L858R-mutated NSCLC whose disease had progressed on or after osimertinib and platinum-based chemotherapy, and with a manageable safety profile [49]. Very recently, the phase III randomized trial named MARIPOSA-2, with 657 randomized patients, has compared two combination regimens, amivantamab plus chemotherapy and amivantamab plus lazertinib plus chemotherapy, versus the standard of care chemotherapy alone, as second-line treatment of advanced EGFR mutant NSCLC progressed on or after treatment with osimertinib [50]. Patients were randomly assigned in a 2:2:1 ratio to receive amivantamab plus lazertinib plus chemotherapy, chemotherapy alone, or amivantamab plus chemotherapy. Amivantamab was administered intravenously but differently from the MARIPOSA trial, with a three-weekly schedule after the initial four weeks of treatment, in order to cycle simultaneously with platin-based chemotherapy. Lazertinib was administered, as usual, orally at 240 mg daily, and the chemotherapy regimen was carboplatin and pemetrexed. The primary endpoint of the study was reached, and both experimental arms, amivantamab plus chemotherapy and amivantamab plus chemotherapy plus lazertinib, produced a statistically significant longer PFS compared with chemotherapy alone (6.3 and 8.3 months for the combinations versus 4.2 months for chemotherapy, HR = 0.48, *p* < 0.001 and HR = 0.44, *p* < 0.001, respectively, for the two comparisons). The PFS benefit, favoring the combinations, was consistent across predefined subgroups. Differently from first-line trials FLAURA 2 and MARIPOSA (where the response rate of standard and experimental arms was similar and the difference came out in terms of duration of response only), as possibly expected, also the antitumoral activity of the two experimental combinations resulted clearly higher in terms of response rate (64% for amivantamab plus chemotherapy, 63% for amivantamab plus lazertinib plus chemotherapy, 36% chemotherapy alone). Follow-up of the trial is too short for conclusions in terms of overall survival, and at the first interim overall survival analysis, the HRs for death were 0.77 (95% CI = 0.49–1.21) for amivantamab plus chemotherapy versus chemotherapy and 0.96 (95% CI = 0.67–1.35) for amivantamab plus lazertinib plus chemotherapy versus chemotherapy.

### 4.2. Safety

Obviously, a price emerged to be paid for the increased efficacy in terms of toxicity. The rate of adverse events of grade 3 or higher, mainly due to hematologic toxicities (mainly thrombocytopenia, neutropenia, and febrile neutropenia), resulted in 72% for patients treated with amivantamab plus chemotherapy, 92% for those treated with amivantamab plus lazertinib plus chemotherapy, and 48% for those treated with chemotherapy alone. However, in terms of the rate of serious adverse events, the combination of amivantamab plus chemotherapy performed better than the triplet and resulted not too far from chemotherapy alone outcomes (32%, 52%, and 20%, respectively). While the rate of infusion-related reactions was similar among the two amivantamab-containing arms (58% and 56% in the doublet and triplet, respectively), the rate of the other amivantamab typically associated adverse events, the venous thromboembolism, was even higher in the triplet arm (22%) than in the doublet one (10%). The rate of discontinuation of all agents due to adverse events was similar among the doublet arm and triplet arm (and similar to those observed for combination regimens of FLAURA 2 and MARIPOSA in first-line setting) and, however, higher than in the chemotherapy-only arm (8%, 10%, 2%, respectively). Hematologic adverse events were also the most common reasons for interruptions, dose reductions, and discontinuations. In conclusion, the MARIPOSA 2 trial is the first phase III randomized trial with two new amivantamab-containing regimens as an alternative, more effective option, compared with chemotherapy alone, as second-line treatment of advanced EGFR mutant NSCLC. Specifically, the combination of amivantamab plus chemotherapy, although more toxic than chemotherapy alone, seems to have a better safety profile in terms of hematologic toxicity compared with the combination of amivantamab plus chemotherapy plus lazertinib, thus having the larger potential of wide clinical use in the future. Obviously, the future application of the MARIPOSA 2 regimens will depend on and will intersect with the application of FLAURA 2 and MARIPOSA regimens in the first-line treatment, with optimal treatment algorithms yet to be defined.

## 5. Subcutaneous Amivantamab

The issue of infusion-related reactions associated with the intravenous administration of amivantamab certainly may negatively influence physicians when they have to choose among the available options for the treatment of advanced EGFR mutant NSCLC, mainly in the first-line setting. Subcutaneous administration of amivantamab is a very interesting option that is being investigated successfully and may strongly reduce the relevance of infusion-related reactions. Moreover, the development of a subcutaneous formulation of amivantamab also aims to improve patient convenience in terms of reducing administration time. Very recently, a phase III randomized trial named PALOMA-3 [51] evaluated the efficacy and safety of subcutaneous amivantamab compared to its intravenous formulation in combination with lazertinib in NSCLC, as third-line treatment of advanced EGFR-mutant NSCLC. Patients whose disease had progressed after osimertinib and platinum-based chemotherapy were randomly assigned 1:1 to receive subcutaneous or intravenous amivantamab, both combined with lazertinib. Among the 418 randomized patients, an objective response rate of 30% was reported for patients treated with subcutaneous amivantamab and 33% for those treated with intravenous amivantamab, and a median PFS of 6.1 and 4.3 months, respectively. Also, in terms of overall survival, the outcome achieved with subcutaneous amivantamab was satisfying (significantly longer than in the intravenous group). As expected, the rate of infusion-related reactions was significantly lower in patients treated with subcutaneous amivantamab versus intravenous (13% versus 66%), and interestingly, also the rate of venous thromboembolism favored the subcutaneous route of administration (9% versus 14%). This is to remind and to underline that the median administration time for the first infusion was 4.8 min for subcutaneous amivantamab and 5 h for intravenous amivantamab. Last but not least, subcutaneous amivantamab achieved non-inferior pharmacokinetics results compared with intravenous amivantamab. These efficacy, safety, and convenience outcomes leave few doubts about an almost complete substitution of intravenous amivantamab with subcutaneous amivantamab in the near future, with an enhancement of the role of this drug in the treatment of advanced EGFR mutant NSCLC (Table 3).

## 6. Antibody-Drug Conjugates

Antibody-drug conjugates (ADCs) are new drugs with an innovative mechanism of action that makes them potentially suitable for the treatment of several solid tumors. In fact, they deliver a cytotoxic agent to tumor cells expressing specific antigens, achieving a better therapeutic index. They have been approved for the treatment of several advanced solid tumors, including recently previously treated metastatic HER2-mutant NSCLC [52]. Datopotamab deruxtecan is an antibody-drug conjugate composed of a humanized trophoblast cell surface antigen 2 (TROP2)–directed monoclonal antibody covalently linked to a topoisomerase I inhibitor payload (deruxtecan). The randomized phase III TROPION-Lung01 study compared the efficacy and safety of datopotamab deruxtecan versus docetaxel in patients with pretreated advanced NSCLC, including patients with actionable genomic alterations such as EGFR mutations [53]. Patients with oncogene-addicted disease had been treated with targeted therapy and with platinum-based chemotherapy combined or not with immunotherapy. About 600 patients were randomized, and 17% of the study population had tumors with actionable genomic alterations, including obviously EGFR-activating mutations. Datopotamab deruxtecan significantly improved PFS versus docetaxel, especially in patients with non-squamous tumors, but did not achieve a statistically significant OS benefit. More specific data for oncogene-addicted disease have been very recently published within the TROPION-Lung05 trial [54]. This phase II study evaluated the safety and activity of datopotamab deruxtecan exclusively in patients with advanced NSCLC whose tumors harbored actionable genomic alterations and progressed on or after targeted therapy and platinum-based chemotherapy. Among 137 patients treated, 57% had EGFR mutations, and the overall response rate in all patients enrolled was 36% and 44% in those with *EGFR* mutations. The data from the TROPION-Lung05 trial, showing very promising antitumor activity in a heavily pretreated EGFR mutant NSCLC population, open the road to further phase III development of datopotamab deruxtecan in this clinical context, with a mechanism of action that goes beyond EGFR pathway.

## 7. Conclusions and Future Direction

As stated by Passaro et al. and Hendriks et al. in recent ESMO consensus panels of experts in lung cancer treatment, first-line third-generation EGFR TKIs such as osimertinib are the current standard of care in the treatment of advanced NSCLC harboring EGFR activating mutations [9,45], with excellent efficacy outcomes and safety profile leading also to relevant quality of life outcomes [10,11]. Beyond osimertinib and lazertinib, other third-generation EGFR TKIs have been developed, such as aumolertinib, furmonertinib, naquotinib, and befotertinib. A recent meta-analysis of randomized trials on these new-generation EGFR TKIs confirmed their superiority compared to first-generation EGFR TKIs in terms of overall survival and antitumor activity, with a different safety profile to be considered [55]. However, resistance to EGFR TKIs, including osimertinib, ultimately always occurs, with systemic and symptomatic progression and death. In fact, although a long treatment period characterized in the majority of patients administered with the third-generation EGFR TKI osimertinib, by brilliant objective responses and clear clinical benefit, also real-world survival data show that the survival rate at 5 years is only 19% in this patient population [56]. Thus, improving first-line treatment outcomes remains an unmet need also for patients with advanced EGFR mutant NSCLC; in fact, it is dismal the evidence that, although an effective treatment such as osimertinib, about 25% of patients do not manage to be treated with a second-line treatment because of death due to lung cancer [57,58]. In the past, other relevant approaches have been tested aimed at improving first-line outcomes for patients with advanced EGFR mutant NSCLC, such as the combination of vascular endothelial growth factor inhibitors with first-generation EGFR TKIs. Two phase III randomized trials have tried to improve the outcomes of the first-generation erlotinib by adding antiangiogenic drugs like bevacizumab or ramucirumab, but only PFS improvements were reported, without statistically significant OS benefits [59,60]. Another option that has been tested in order to improve the outcomes of second-line treatment of advanced EGFR mutant NSCLC after EGFR TKI therapy is the four-drug combination of immunotherapy, antiangiogenic therapy, and platin-based chemotherapy. An exploratory subgroup analysis of the IMpower150 phase III randomized trial had shown an overall survival benefit in favor of carboplatin plus paclitaxel plus bevacizumab plus atezolizumab versus carboplatin plus paclitaxel plus bevacizumab in the subgroup of patients enrolled in the trial with EGFR mutant advanced NSCLC pretreated or not with EGFR TKIs [61]. As a consequence, a phase III randomized trial specifically compared the four-drug combination with chemotherapy alone (cisplatin or carboplatin plus pemetrexed) in EGFR mutant or ALK translocated NSCLC that progressed after TKIs therapy and advantages in terms of response rates and PFS were reported in favor of the immunotherapy containing regimen (increasing as PDL-1 expression increased) but not in terms of OS [62]. Thus, new treatment options were eagerly awaited and have been recently reported in FLAURA 2 and MARIPOSA trials, with very interesting results that improve or at least widen the spectrum of first- and second-line treatment options of this peculiar patient population. As seen above, the combination of chemotherapy plus osimertinib has achieved an improvement in progression-free survival and is an option, especially for young patients with unfavorable prognostic factors (such as patients with brain metastases or L858R mutations), in order to reduce the risk of early progressive disease, at the cost of worse safety profile compared to osimertinib alone [18]. The combination of the bi-specific anti-EGFR and anti-MET inhibitor amivantamab with the third-generation EGFR TKI lazertinib has recently been established as another valuable option to improve progression-free survival outcomes in this patient population compared to osimertinib alone [19]. Also, this combination has a more complex safety profile compared to osimertinib alone, especially in terms of infusive reactions, however significantly reduced with corticosteroids premedication and, in particular, with subcutaneous formulation, and thrombus venous embolism, but, as chemotherapy plus osimertinib, it should be considered a potentially more effective option, compared to osimertinib alone that should always be discussed with patients, especially young ones, in order to produce a real shared decision making. In the near future, we may have the possibility to choose among three first-line treatment options for our patients with advanced EGFR mutant NSCLC, thus having the challenge to personalize treatments. We may prefer the option of osimertinib alone for elderly patients (i.e., older than 75 years), for patients with several and/or severe comorbidities not suitable for more toxic combinations, for patients without any of the recognized poor prognostic factors (only thoracic disease and exon 19 deletion alone) and for all patients that however express the will to be treated with an all-oral and less toxic regimen although the possibility that an intravenous combination regimen may produce a superior efficacy outcome in their disease. We could speculate and may prefer the combination of platin-based chemotherapy and osimertinib in young patients without severe comorbidities whose tumors present with brain metastases and/or L858R mutation. Or we may prefer the combination of amivantamab plus lazertinib in patients with liver metastases and/or TP53 co-mutations and in all young and fit patients that express the will to exert a proactive action against the emergence of resistance to EGFR-TKI and that do not want to be administered with chemotherapy. However, all these kinds of criteria can be considered speculative and not clearly evidence-based. Moreover, when choosing among available first-line treatment options, the discussion with the patient aimed at real shared decision-making cannot preclude a description of the toxicity profiles of the two new available combinations. In conclusion, the availability of chemotherapy plus osimertinib and amivantamab plus lazertinib in the first-line treatment and amivantamab plus chemotherapy in the second-line treatment gives physicians and patients a significantly wider spectrum of therapeutic options in this clinical setting. However, the multiple options of first- and second-line treatment should be considered at the beginning of the therapeutic patient journey. To date, a standard treatment algorithm can not exist because the algorithm should be individualized, considering multiple factors such as age, performance status, comorbidities, disease sites, molecular characteristics, safety profiles of treatment options, and, last but not least, patient preference. However, in the near future, definitive overall survival data of FLAURA 2 and MARIPOSA trials may be reported and possibly change our views and give more precise suggestions on the first- and, as a consequence, also on the second-line standard treatments of advanced EGFR mutant NSCLC.

## Figures and Tables

**Table 1:** Efficacy and safety outcomes (phase III randomized trials) of osimertinib plus platin-based chemotherapy (FLAURA 2) and of amivantamab plus lazertinib (MARIPOSA) compared with osimertinib alone in the first-line treatment of advanced EGFR mutant NSCLC.

Regimen/Trial	RR/Median Duration of Response	Median PFS	OS	AE ≥ Grade 3	Rate of Discontinuation of All Agents
**Osimertinib/FLAURA 2**	76%/15.3 months	16.7 months	Survival rate at 24 months 73%	27%	6%
**Osimertinib plus platin-based chemotherapy** **/FLAURA 2**	83%/24 months	25.5 months (HR vs. OSI = 0.62; *p* < 0.001)	Survival rate at 24 months 79% (HR = 0.90; *p* = 0.52	64%	11%
**Osimertinib** **/MARIPOSA**	85%/16.8 months	16.6 months	Survival rate at 24 months 69%	43%	3%
**Amivantamab-lazertinib** **/MARIPOSA**	86%/25.8 months	23.7 months (HR vs. OSI = 0.70 *p* < 0.001)	Survival rate at 24 months 74% (HR = 0.80; 95% CI, 0.61 to 1.05)	75%	10%

RR: response rate; PFS: progression-free survival; OS: overall survival; AE: adverse events; HR: hazard ratio; CI: confidence intervals.

**Table 2:** Efficacy and safety outcomes of amivantamab combined with platin-based chemotherapy and with platin-based chemotherapy plus lazertinib, compared with chemotherapy alone in the second-line treatment of advanced EGFR mutant NSCLC.

Regimen/Trial	RR/Median Response Duration	Median PFS	OS	AE ≥ Grade 3	Rate of Discontinuation of All Agents
**Platin-based chemotherapy** **/MARIPOSA 2**	36% /5.6 months	4.2 months		48%	2%
**Amivantamab plus platin-based chemotherapy** **/MARIPOSA 2**	64% /6.9 months	6.3 months HR vs. CT = 0.48, *p* < 0.001	HR vs. CT = 0.77 *p* = 0.25	72%	8%
**Amivantamab plus platin-based chemotherapy plus lazertinib** **/MARIPOSA 2**	63% /9.4 months	8.3 months (HR vs. CT = 0.44, *p* < 0.001)	HR vs. CT = 0.96 *p* = 0.80	92%	10%

RR: response rate; PFS: progression-free survival; OS: overall survival; AE: adverse events; HR: hazard ratio; CT: chemotherapy.

**Table 3:** The main safety outcomes of the amivantamab plus lazertinib combination were found in the MARIPOSA and PALOMA 3 trials (with intravenous and subcutaneous amivantamab, respectively).

Regimen/Trial	AE ≥ Grade 3	Rate of Infusion-Related Reactions	Rate of Venous Thromboembolism	Rate of Discontinuation of All Agents
**Amivantamab plus lazertinib/MARIPOSA (first-line setting)**	**75%**	**63%**	**37%**	**10%**
**Amivantamab plus lazertinib/PALOMA 3 (third-line setting)**	**52%**	**13%**	**9%**	**9%**

AE: adverse events.
